# GazeParser: an open-source and multiplatform library for low-cost eye tracking and analysis

**DOI:** 10.3758/s13428-012-0286-x

**Published:** 2012-12-13

**Authors:** Hiroyuki Sogo

**Affiliations:** Ehime University, 3 Bunkyo-cho, Matsuyama, Ehime 790-8577 Japan

**Keywords:** Eye tracking, Open source, Python

## Abstract

Eye movement analysis is an effective method for research on visual perception and cognition. However, recordings of eye movements present practical difficulties related to the cost of the recording devices and the programming of device controls for use in experiments. GazeParser is an open-source library for low-cost eye tracking and data analysis; it consists of a video-based eyetracker and libraries for data recording and analysis. The libraries are written in Python and can be used in conjunction with PsychoPy and VisionEgg experimental control libraries. Three eye movement experiments are reported on performance tests of GazeParser. These showed that the means and standard deviations for errors in sampling intervals were less than 1 ms. Spatial accuracy ranged from 0.7° to 1.2°, depending on participant. In gap/overlap tasks and antisaccade tasks, the latency and amplitude of the saccades detected by GazeParser agreed with those detected by a commercial eyetracker. These results showed that the GazeParser demonstrates adequate performance for use in psychological experiments.

## Open-source library for low-cost eye tracking and analysis

For the last several decades, eye movement measurement has been consistently used in research on visual perception and cognition in such areas as space perception, scene recognition, reading, spoken language processing, and clinical studies (van Gompbel, Fischer, Murray, & Hill, 2007). Although eye movement measurement is an effective method for these research areas, there remain certain barriers that restrict its application in other research areas. One of these barriers is the cost of eyetrackers—that is, eye movement recording devices. Some commercial eyetrackers that are used in eye movement research cost in excess of $40,000. Recently, several low-cost open-source eyetrackers have been developed mainly for use as human–computer interaction devices (Li, Babcock, & Parkhurst, 2006; San Agustin, Skovsgaard, Mollenbach, Barret, Tall, Hansen, & Hansen, 2010; Zielinski, 2007). For example, the ITU Gaze Tracker (San Agustin et al., 2010) performs real-time eye movement measurement from ocular images taken from a standard USB Web camera or a video camera with night vision. Although the spatial resolution of these eyetrackers is adequate for research on visual perception and cognition, the sampling frequency of eye movements is limited to the camera’s speed. The majority of camera units that are adaptable to such open-source eyetrackers capture, at most, 30 frames per second. This means that the sampling rate of eye position is only 30 Hz. Although this sampling frequency is sufficient for achieving human–computer interactions, research on visual perception and cognition sometimes requires much higher sampling frequencies.

A second barrier in research applications of these eye movement measurement tools is the difficulty in achieving eyetracker synchronizations with stimulus presentations. Some commercial eyetrackers have libraries that provide methods for synchronizing the eyetracker in conjunction with a stimulus presentation program. For example, the Eyelink (SR Research Ltd.) eyetracker is easily controlled from Python scripts using the Pylink package, provided by the manufacturer. Using Pylink packages with Python-based experimental control libraries, such as VisionEgg (Straw, 2008) and PsychoPy (Peirce, 2007), an experimenter can start and stop the recording of eye movement with precise timing. Similarly, Eyelink can be easily controlled from MATLAB scripts using Psychophysics Toolbox (Brainard, 1997; Pelli, 1997) in conjunction with Eyelink Toolbox (Cornelissen, Peters, & Palmer, 2002). However, because current open-source eyetrackers do not have such libraries, researchers must write programs to synchronize an eyetracker with experimental presentations if they use open-source eyetrackers; this creates difficulty in the use of open-source eyetrackers for research.

In the present article, I describe the use of an open-source eye-tracking library, GazeParser. This tracking library consists of two components. The first component is an application that captures ocular images from a camera to record eye position. The second component is a Python library for calibrating, synchronizing stimulus presentation and recording, and analyzing eye movements. GazeParser relies on Python packages such as OpenCV (Bradski, 2000), SciPy (Jones, Oliphant, & Peterson, 2001), and Matplotlib (Hunter, 2007) for camera image analysis and data visualization. VisionEgg or PsychoPy can be used with GazeParser for stimulus presentation. Although GazeParser is designed mainly for use with Microsoft Windows, a cross-platform edition is currently under development for use not only with Windows, but also with Linux and Mac OS X operating systems. This software is distributed through the GazeParser project page (http://gazeparser.sourceforge.net/); documents pertaining to the use of the software are also available there.

In the present study, three experiments were conducted to evaluate performance of the GazeParser library. In the first experiment, eye movement during fixation on a small square was recorded to evaluate the temporal and spatial characteristics of GazeParser. In the second experiment, temporal accuracy and precision were examined when sampling frequency was increased to 500 Hz. In the third experiment, eye movements during performance of an antisaccade task (Everling & Fischer, 1998) and a gap/overlap task (Fischer & Weber, 1993) were recorded to test the performance of the GazeParser in a condition similar to those in actual eye movement research.

## General method

### Apparatus

Two Windows personal computers (PCs) were used. One recorded eye movements (*recorder* PC), and the other was used to present visual stimuli to participants (*presentation* PC). A camera unit was connected to the recorder PC to capture ocular images. Different recorder PCs and camera units were used for the three experiments; details are described in the Method section for each experiment. A LAN cable connected the recording and presentation PCs. A 22.5-in. liquid crystal display (LCD) was connected to the presentation PC. Experiments were controlled by a Python script run on the presentation PC. The VisionEgg library was used for displaying visual stimuli.

Each experiment was performed in a dimly lit room. A participant sat on a chair; a head- and chinrest restricted head movement. The LCD was placed at a distance of 57 cm from the participant for stimulus presentation. The refresh rate of the LCD was 60 Hz. A keyboard situated in front of the participant was connected to the presentation PC.

The camera unit was placed in front of a participant’s left eye, with the vertical level of the camera lower than this eye. This alignment ensured that the camera would not disturb a participant’s view of the LCD. Distance between the camera and the participant was adjusted for each participant so that the participant’s left eye was captured full in the camera image; typically, this distance was about 15 cm. An infrared (IR) LED light was placed just below the LCD to illuminate the participant’s eye.

### Synchronizing recording and presentation PCs

The recording and presentation PCs sent commands and received data though a TCP/IP connection. TCP “NO_DELAY” mode was used to minimize the delay for sending commands. Calibration and recoding processes on the recorder PC were controlled from the presentation PC. Information on events during the experiment, such as the onset time of a visual stimulus and keypresses of the participant, were sent from the presentation PC to the recorder PC. The recorder PC collected gaze position data and information on these events, which were stored in a CSV (comma-separated values) format file.

### Getting gaze position

The pupil of a participant’s eye and the reflection image of the IR light from the cornea (the Purkinje image) were detected each time a new camera image was captured. Here *U* and *V* denote the center of the Purkinje image, relative to the center of the pupil, in the camera image coordinate. The gaze position on the screen was calculated using the following equation:

$$ \left( {X,Y} \right)=\left( {a_X U+{b_X}V+{c_X},{a_Y}U+{b_Y}V+{c_Y}} \right). $$

*X* and *Y* denote horizontal and vertical gaze positions on the display coordinate. The *a*_*X*_, *b*_*X*_, *c*_*X*_, *a*_*Y*_, *b*_*Y*_, and *c*_*Y*_ terms are parameters to be estimated from calibration data. In the calibration procedure, (*U, V*) were collected while the participant fixated on a target, which was sequentially presented at several locations. The arrangement of target locations can be customizable. The target was presented for 1 s per position. Following this, parameters were estimated by finding numerical values that minimized the sum of square distances between the gaze position and the target position. To avoid including eye movement changes in the fixation point in the calibration data, data that were obtained within 200 ms before and after the target had moved to a new position were discarded.

### Saccade detection

Detection of saccades and fixations was performed offline. The detection algorithm had three parameters: “VelocityThreshold,” “MinimumSaccadeDuration,” and “MinimumFixationDuration.” At the beginning of the detection process, eye movements that exceeded the VelocityThreshold were extracted. Movements that were continuously faster than VelocityThreshold and longer than MinimumSaccadeDuration were marked as saccade candidates. If the interval between successive saccade candidates was shorter than MinimumFixationDuration, these saccade candidates were merged together. After these processes were completed, saccade candidates were considered to be saccades, and the intervals between these saccades were considered to be fixations.

In the present study, detection of saccades and fixations was performed only in Experiment 3 (see the Method section of Experiment 3 for relevant parameters).

### Recording and data analysis with GazeParser

**Fig. 1:**
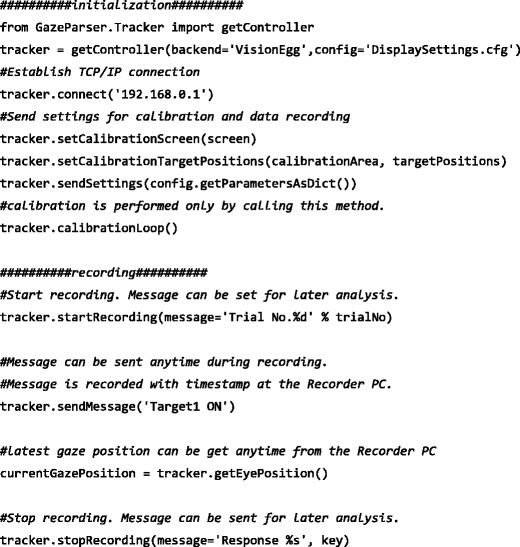
An example of experiment script with GazeParser. Only important portions are shown

Figure 1 shows an example of an experimental script. At the beginning of the experiment script, written with VisionEgg or PsychoPy, a few commands were inserted to initialize GazeParser (i.e., the “initialization” portion of Fig. 1); these included IP address of the recorder PC, location of calibration target, and other settings such as viewing distance. Adjustment of camera angle and parameters, performing calibration, and verifying calibration results could be done only by calling the calibrationLoop() method.

At the beginning of each trial, the startRecording() method was called to start data recording, and at the end of each trial, the stopRecording() method was called to stop recording. By passing messages through these methods, information on each trial and the gaze position data could be collated and saved together. During recording, an event such as a stimulus onset was saved with a timestamp by sending a message using the sendMessage() method. The latest gaze position was available by calling the getEyePosition() method.

Data were recorded at the recorder PC as a CSV format file. Saccades and fixations were detected from this CSV file and saved as “GazeData” object, using GazeParser’s data conversion module. Parameters such as latency of saccade relative to an event and duration of fixation are readily calculated using class methods of the GazeData objects. For example, the saccade following a given message can be found by using the getNextEvent() method. The latency of a saccade relative to a given message can be obtained by the relativeStartTime() method. Detailed documents of these Python modules and methods are available at the GazeParser project page (http://gazeparser.sourceforge.net/).

## Experiment 1

To evaluate performance of the GazeParser, eye movements were recorded while participants fixated on a small square presented at randomly selected locations on a computer display. The primary aim of Experiment 1 was to test effects of the recorder PC on the temporal accuracy and precision of the data, using two recorder PCs.

### Method

#### Participants

Five volunteers and the author participated in the experiment. All volunteers were naïve as to eye movement research. All participants had normal or corrected-to-normal vision. None had any type of oculomotor dysfunction.

#### Apparatus

Two configurations of eye-tracking hardware, hardware sets 1a and 1b, were used (Table 1). Hardware set 1a is a sample of a low-price PC that has adequate spec for office work, such as document writing and Web browsing. Hardware set 1b is a sample of a more powerful PC, as compared with hardware set 1a. In hardware set 1a, a laptop PC (Intel Core i3 M370 CPU, HM55 express chipset) was used for the recorder PC. An OptiTrack V120slim (Naturalpoint Inc.) camera was used to capture ocular images; the frame rate of the camera was set to 120 Hz. The size of the camera image was set to 320 × 240, and the camera unit was connected to the recorder PC by USB2.0 interface. In configuration 1b, a desktop PC (Intel Core i7 950 CPU, Intel X58 chipset) was used as the recorder PC. The camera unit was the same as that in hardware set 1a.

**Table 1:** Hardware setup

	Set 1a	Set 1b	Set 2
Recorder PC			
CPU	Intel Core i3 M370	Intel Core i7 950	Intel Core i7 950
Chipset	Intel HM55	Intel X58	Intel X58
Camera			
Camera unit	OptiTrack V120slim	OptiTrack V120slim	IMPREX ICL-B0620
Interface	USB2.0 (On board)	USB2.0 (On board)	Interface PEX-530421

#### Procedure

Participants sat in the chair, and the experimenter adjusted the position of the headrest, chinrest, and camera. After confirming that the pupil and the Purkinje image were correctly detected, calibration was initiated. The calibration period began with the presentation of a target in the form of a black square (0.26° × 0.26° in visual angle) in the center of the screen. When a participant pressed the space key, the target moved to one node of a 3 × 3 invisible grid. The center of the grid was located at the screen center, and the horizontal and vertical distances between adjoining grid nodes were 9.3° and 6.6°, respectively. Participants were instructed to move their eyes to track the target motion. The target was steadily moved to a new location over a period of 1,000 ms; it then remained at that location for 1,000 ms. The center of the Purkinje image relative to the pupil center was corrected for calibration within a period of 200–800 ms after the target arrived at a new location. The calibration process was completed when the target sequentially visited each of the nine locations once. The order of visiting these locations was randomly determined.

Following the calibration period, experimental trials were presented. The first experimental trial began with the presentation of a target smaller (0.13° × 0.13° black square) than that used in calibrations. After a participant pressed the space key, the target jumped to a location that was randomly selected from the nodes of a 6 × 6 invisible grid. The center of this grid was located at the screen center. The horizontal and vertical distances between adjoining grid nodes were 2.7°. Note that no grid node position corresponded to calibration target positions. Participants were instructed to make a saccade to the target when it jumped from one location to another. The target stayed at one node for 1,000 ms and then jumped to an unvisited node. Each trial was completed 1 s after the target jumped to the 10th node. The recorder PC recorded gaze position from the beginning to the end of all trials. Participants performed 20 trials per each hardware set; they could take a break between trials without moving their head off the head- and chinrest.

Prior to each time a new screen was drawn on the presentation PC, this computer transferred a request to the recorder PC to send the latest recorded gaze position back to the presentation PC. Timestamps indicating when requests were sent and data received were recorded on the presentation PC, and this PC also requested the recorder PC to record the time at which a target jumped to a new location.

#### Data analysis

Gaze position data within 250–750 ms after the time when the target jumped to a new position were used to estimate spatial accuracy and precision. The saccade detection feature of the GazeParser was not used in data analysis of this experiment.

### Results and discussion

#### Temporal accuracy and precision

**Fig. 2:**
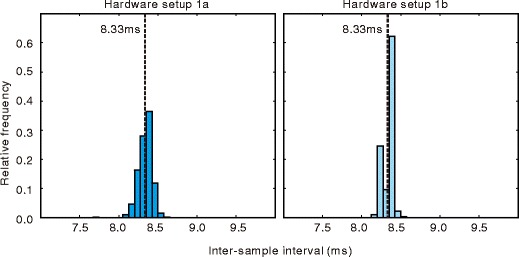
Distribution of intersample interval. *Vertical dashed lines* indicate the ideal interval (8.33 ms)

To evaluate the effects of the performance of the recorder PC on the temporal accuracy of data sampling, intersample intervals were calculated for the data recorded by hardware sets 1a and 1b. The data for all participants were collapsed. Figure 2 shows the frequency distribution of intersample intervals obtained from these hardware sets. Table 2 shows the mean, standard deviation (*SD*), 0.5 percentile point, and 99.5 percentile point, as well as the minimum and maximum values of intersample intervals. Because the frame rate of the camera was 120 Hz, the ideal intersample interval was 8.33 ms. In both hardware sets, the mean interval was close to this ideal value. As compared with the intersample intervals recorded by hardware set 1b, those recorded by hardware set 1a were broadly distributed. It is noteworthy that the maximum intersample interval of 29.8 ms was greater than 16.67 ms—that is, the double of the ideal intersample interval. The likely cause of this was that the recorder PC could not follow the camera’s sampling speed. Intersample intervals longer than 16.67 ms were observed four times among all data recorded by hardware set 1a. On the other hand, the maximum intersample interval was 8.60 in the data obtained by hardware set 1b.

**Table 2:** Temporal accuracy and precision in Experiment 1

	Hardware set 1a	Hardware set 1b
Mean	8.34	8.34
Standard deviation	0.54	0.06
0.5 percentile point	8.07	8.17
99.5 percentile point	8.55	8.48
Minimum interval	0. 77	8.01
Maximum interval	29.7	8.60

Unit: milliseconds

**Fig. 3:**
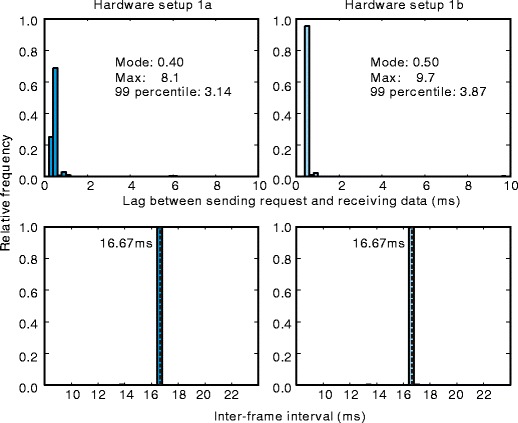
Temporal performance of inter-PC communication. *Top row*: Distribution of the lag between sending a request from the presentation PC and receiving gaze position data from the recorder PC. *Bottom row*: Intervals between requests for current gaze position. The ideal interval was equal to the interframe interval of the LCD (16.67 ms, indicated by a vertical dashed line)

The time lag in communicating between the presentation PC and recorder PC is also an important point for evaluating temporal performance. In this experiment, a long lag might cause skipping of video frames because the presentation PC stopped drawing new frames until the latest gaze position was sent back from the recorder PC. To evaluate the time lag, the distribution of the time lags between sending a request from the presentation PC and receiving the gaze position data from the recorder PC (top row of Fig. 3) was analyzed. The peak of this distribution was shorter than 1 ms, and 99 % of the lag times were shorter than 4 ms. The maximum lag was 8.1 ms for hardware set 1a and 9.7 ms for hardware set 1b. These values were shorter than the interframe interval of the LCD (16.67 ms). To ensure that frame skipping did not occur, I analyzed the distribution of intervals between successive sendings of requests. Because requests were sent immediately after every drawing frame, this interval was equal to the interframe interval. The bottom row of Fig. 3 shows the distribution of these requests. All request intervals were within 1.0 ms from the ideal interval of 16.67 ms. This is consistent with the conclusion that no frame skipping occurred in the present data.

In summary, the difference between the mean intersample time interval and the ideal interval was less than 0.1 ms in both hardware setups tested in Experiment 1. The present findings confirm that temporal accuracy was adequate for research on eye movements. Ninety-nine percent of intersample intervals fell within approximately ±0.3 ms from the ideal interval; however, several samples were lost when the data were recorded by a PC that was not designed for lab use (hardware set 1a). To prevent the loss of samples, a higher quality PC is necessary. Communication between the recorder PC and presentation PC was rapid enough to transfer current gaze position prior to each time stimuli were drawn on the presentation PC at the refresh rate of 60 Hz.

#### Spatial accuracy and precision

**Fig. 4:**
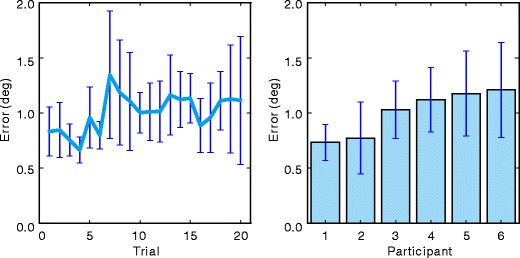
Mean distance between target position and gaze position (spatial error). *Left panel*: Effects of time spent from calibration on the spatial error. *Right panel*: Individual differences in the spatial error. In both charts, spatial error was averaged over all target positions

To evaluate spatial accuracy and precision, the mean distance between the target position and recorded gaze position was calculated. For simplicity, this value is referred to as *spatial error* in this article. The left panel of Fig. 4 shows the spatial error calculated trial by trial. The data for all target positions on a single trial were collapsed. A repeated measures one-way analysis on variance (ANOVA) showed that the effect of trial number on mean distance was significant, $F\left( {19,95} \right)=1.80,p=.03,\eta_p^2=.26$. Post hoc comparison with Bonferroni correction showed that only the spatial errors in trial 4 and trial 7 were significantly different (*p* < .05). Although minor head movements after calibration may potentially cause a decrease in spatial accuracy and precision, such a decrease was not prominent in this experiment.

The right panel of Fig. 4 shows the mean spatial error calculated for each participant. The data for all target positions on all trials were collapsed. The mean spatial error ranged from 0.7° to 1.2°. Considering that spatial errors of research and commercial video-based eyetrackers range from 0.5° to 4.0° (Hansen & Ji, 2010), it can be considered that spatial accuracy of GazeParser falls within an acceptable range for use as a video-based eyetracker. A spatial error of 0.7°–1.2° would be problematic for research in which high spatial accuracy is required; for example, the particular letter that is fixated on in a word must be strictly distinguished in research on sentence reading. However, there would be much research in which this amount of spatial error is acceptable.

## Experiment 2

In Experiment 2, the sampling frequency of eye tracking was boosted in order to examine the upper limits of sampling frequency recording possible with the GazeParser system. That is, high-frequency recording presents two difficulties. First, the intersample interval becomes too brief at very high frequencies for the recorder PC to process camera images during recording. Second, saccade detection can increase in difficulty at higher frequencies due to low signal–noise ratios. As sampling frequency increases, the amount of eye movement between subsequent samples decreases if the eye moves at the same velocity. Additionally, the shutter speed of the camera becomes faster as sampling frequency increases, and this causes greater noise in the camera image. Therefore, as sampling frequency increases, the signal (eye movement) decreases, and image noise increases. To examine performance of the GazeParser during high-frequency recording, data were recorded at 250, 400, and 500 Hz.

### Method

#### Participants

The author participated in this experiment.

#### Apparatus

Hardware set 2 (Table 1) was used to record data. The recorder PC was the same PC as that used with hardware set 1b. A Bobcat ICL-B0620 (IMPREX Inc.) camera unit was used to capture ocular images. An IR pass filter (cutoff frequency = 800 nm) was inserted between camera and lens to reduce noise. The ICL-B0620 was connected to the recorder PC through an image grabber PEX-530421 (Interface Corp.). The frame rate of this camera is 260 Hz at its default setting; however, to capture images at higher frame rate, the camera was set to run at 2 × 2 binning, overclock mode, 320 × 224 pixels of image size. As a result, the frame rate of the camera was boosted to 500 Hz. In this experiment, the operation of the system was tested at sampling rate of 250, 400, and 500 Hz.

#### Procedure

The calibration procedure and the task were the same as those in Experiment 1. Participant performed 20 trials for each camera frame rate (250, 400, 500 Hz).

#### Data analysis

To examine the effect of low-pass filtering on saccade detection, a zero-phase third-order Butterworth low-pass filter (Gustafsson, 1996) was applied to the gaze position during offline analysis. The cutoff frequency of the filter was 60 Hz. Data obtained in Experiment 1 (120 Hz of sampling rate) were used for comparison. Because data sampled at 120 Hz contain no frequency components higher than the Nyquist frequency (i.e., 60 Hz), a low-pass filter was not applied to the data from Experiment 1.

### Results and discussion

**Fig. 5:**
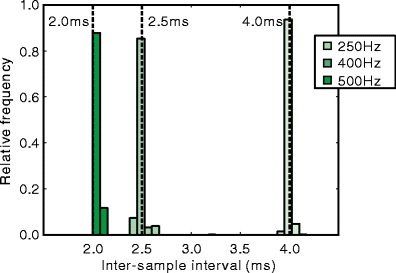
Distribution of the intersample interval in Experiment 2. *Vertical dashed lines* indicate the ideal interval

Figure 5 shows the intersample intervals in 250-, 400-, and 500-Hz measurements. In all measurements, the center of the distributions was close to the ideal value. Table 3 shows the mean, *SD*, 0.5 percentile point, and 99.5 percentile point, as well as the minimum and maximum of the intersample intervals. The difference between the mean intersample interval and an ideal interval was less than 0.01 ms. On the other hand, the maximum interval reached 4.0 ms in all measurement conditions. Because 4.0 ms is twice as long as the ideal interval for the 500-Hz measurement, this result implies that data loss might occur often in 500-Hz measurement. Although the camera was capable of higher-frequency image capturing, 500 Hz should be the limit of the current hardware set.

**Table 3:** Temporal accuracy and precision in Experiment 2

	250 Hz	400 Hz	500 Hz
Ideal interval	4.00	2.50	2.00
Mean	4.00	2.50	2.00
Standard deviation	0.02	0.05	0.08
0.5 percentile point	3.93	2.40	1.83
99.5 percentile point	4.07	2.60	2.12
Minimum interval	3.89	1.10	0.44
Maximum interval	4.12	4.00	4.06

Unit: milliseconds

**Fig. 6:**
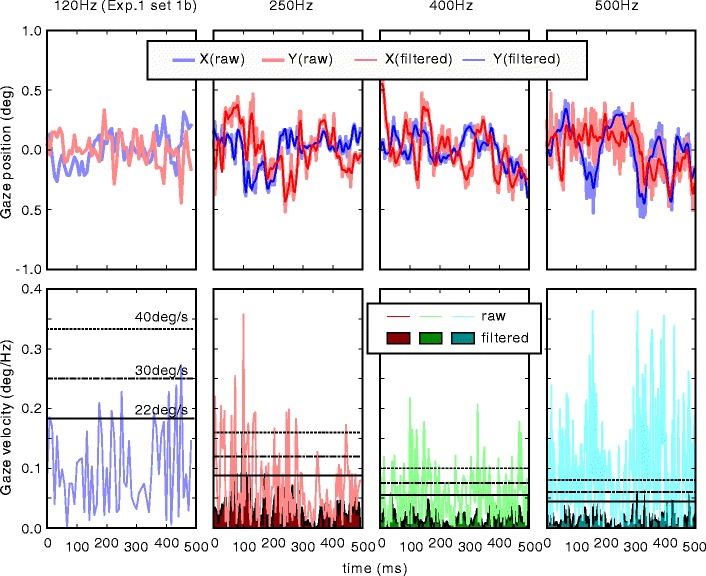
Effect of measurement noise on detecting saccades. Columns correspond to 120-, 250-, 400-, and 500-Hz measurements from left to right. *Top row*: Raw data and low-pass filtered data (only raw data are plotted for the 120-Hz measurement). *Bottom row*: Absolute velocity of raw data and filtered data. Note that the unit is not °/s but °/Hz. Horizontal black lines indicate 40, 30, and 22 °/s from top to bottom

Figure 6 shows the effect of measurement noise on saccade detection. The top row of Fig. 6 shows examples of the recorded gaze position while fixated on a target for 500 ms. The thick pale lines represent raw data, and the thin vivid lines represent filtered data. The bottom row of Fig. 6 shows absolute velocity of the gaze position. The pale lines and dark areas represent absolute velocity calculated from raw data and filtered data, respectively; horizontal black lines in the bottom row indicate 22, 30, and 40 °/s from bottom to top. A velocity of 22 °/s was used as the threshold for saccade detection in the next experiment (Experiment 3). Velocities of about 20–40 °/s are usually used as threshold when saccade is defined as an eye movement faster than a threshold value. Measurement noise was so large that raw velocity fluently exceeded these threshold values. By contrast, low-pass filtered velocity scarcely exceeded the thresholds when compared with raw velocity. These occasional “breakthroughs” of threshold are unlikely to be judged as saccades if the minimum duration of a saccade is set appropriately. A drawback of using a low-pass filter is that high-frequency components of minute eye movements, such as tremors with frequencies close to 80 Hz (Findlay, 1971), might be distorted or lost by low-pass filtering.

In summary, the hardware set tested in this experiment could effectively manage sampling frequencies of up to 500 Hz. However, conservative strategy would entail limiting the sampling frequency to 400 Hz or less to avoid data loss. Applying a low-pass filter would be necessary for high-frequency recording to detect saccades based on velocity threshold.

## Experiment 3

In Experiment 3, saccades were detected from data recorded while participants performed both an antisaccade task and a gap/overlap task. The goal was to examine the performance of GazeParser’s saccade detection function. A commercial eyetracker, Eyelink, was also used to record and detect saccades for comparison.

### Method

#### Participants

Five naïve volunteers and the author participated in the experiment. One of the 5 naïve volunteers and the author had also participated in Experiment 1. All participants had normal or corrected-to-normal vision. None had any type of oculomotor dysfunction.

#### Apparatus

Hardware set 1b in Experiment 1 was used to record eye movements. For comparison, an Eyelink eyetracker was also used to record eye movements. The Eyelink was connected to the presentation PC using a LAN cable. The Pylink library (SR Research Ltd.) was used to control Eyelink from the presentation PC.

#### Procedure

The experiment consisted of an antisaccade task and a gap/overlap task. The antisaccade task consisted of four blocks, two *antisaccade* blocks, and two *prosaccade* blocks. At the beginning of each block, a nine-point calibration was performed. During calibration, participants fixated on a small black square that appeared sequentially at nine locations on the screen. The camera was adjusted, and participants performed the calibration task until the measurement error was less than 0.8° for the average of the nine locations. Upon completion of calibration, a message appeared instructing participants as to whether they should perform an antisaccade or a prosaccade task in the following block. The first experimental trial of each block began immediately after participants read the (above) message and pressed the space key. At the beginning of each trial, an initial fixation point (a 0.33° × 0.33° square) was presented at the center of the screen. The color of this fixation square was red in the antisaccade block and green in the prosaccade block. The duration of this fixation square was 1,000, 1,100, 1,200, 1,300, 1,400, or 1,500 ms. Immediately after the fixation point disappeared, a target (a 0.33° × 0.33° white square) was presented above, beneath, to the left, or to the right of the location of the initial fixation point. The distance between the position previously occupied by the fixation point and the target was 4.2°. Participants were instructed to make a saccade to the direction opposite of the target in the antisaccade block (i.e., if the target appeared below the fixation point, they should make an upward saccade from the point indicated by the fixation stimulus), whereas they were instructed to make a saccade *to* the target in the prosaccade block. Three seconds after the onset of the initial fixation point, the trial ended, and the target disappeared. The next trial started automatically after a 500-ms interval from the end of the trial. Twenty trials were performed in each block. The presentation duration of the initial fixation point was randomly decided on each trial, and the direction of the target was balanced within each block.

The procedure of the gap/overlap task was the same as that of the antisaccade task, except for the following. Participants were instructed to make a saccade to the target in all four blocks. The color of the initial fixation point was white in all blocks. The target appeared either to the left or to the right of the initial fixation. The gap duration—that is, onset time of the target relative to the offset of the initial fixation point—was −200, −100, 0, 100, or 200 ms. Negative gap duration indicated that the initial fixation point disappeared before the onset of the target, and the gap duration was balanced within each block.

Each participant performed the antisaccade task and the gap/overlap task once, with eye movements recorded by the GazeParser and once by the Eyelink. The order of the measurement devices was balanced between participants.

#### Data analysis

Parameters for saccade detection are shown in Table 4. A velocity threshold of 22.0 °/s and an acceleration threshold of 4,000 °/s^2^ for Eyelink was a typical configuration for a psychophysical experiment according to the Eyelink user manual. A low-pass filter was not applied to gaze position data.

**Table 4:** Parameters for saccade detection in Experiment 3

	GazeParser	Eyelink
Velocity threshold	22 °/s	22 °/s
Acceleration threshold	*	4,000 °/s^2^
Minimum saccade duration	12 ms	*
Minimum fixation duration	12 ms	*

* No corresponding parameter

The first saccade executed after onset of the target was used for analysis. If the onset time of the first saccade relative to the target onset (i.e., latency) occurred within a range of 100–600 ms, it was considered that the participant had made a saccade correctly on that trial. Trials on which the first saccade did not land on a point further than 2.0° from the target position were also considered saccade mistakes. These error trials were removed from further analyses.

### Results and discussion

**Fig. 7:**
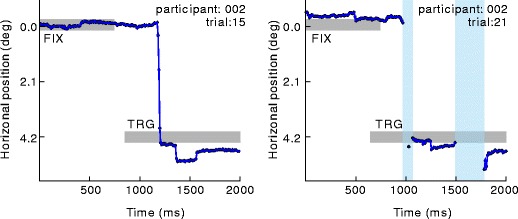
Examples of recorded data. *Left and right panels* show data from the same participant. *Blue lines* indicate gaze position, and gray bars indicate presence of the initial fixation point and the target. *Pale blue areas* indicate that eye position detection failed during this period. In the left panel, eye position detection failure occurred near the saccade onset; therefore, that detection of saccade onset failed. Such a failure occurred in 2 participants more often, when compared with the other participants

Regarding the data recorded by Eyelink, 6.3 % (*SD* = 1.3 %) of the trials were removed, on average. On the other hand, 15.5 % (*SD* = 9.8 %) of the trials were removed from the data recorded by GazeParser. This difference resulted from occasional failure of eye position detection in 2 participants (Fig. 7) who did not serve in Experiment 1. When the data of these 2 participants were removed (32 % and 26 %, respectively) from analysis, the ratio of removed trials decreased to 8.8 % (*SD* = 2.8 %). Because there was no problem in saccade detection concerning trials with successful eye position detection, the data of these participants were used in the following analyses.

**Fig. 8:**
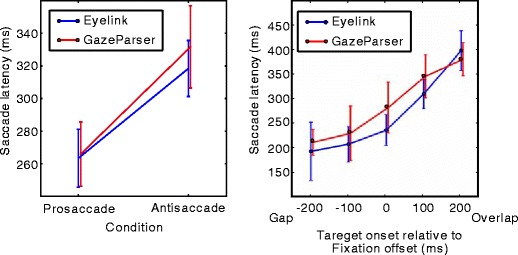
Mean saccade latencies in the antisaccade task and the gap/overlap task. The results obtained by GazeParser agreed with those obtained by Eyelink

Figure 8 shows the mean saccade latency in the antisaccade task and the gap/overlap task, collapsed across all saccade directions. As previous studies have shown, the mean latency in the antisaccade condition was longer than that in the prosaccade condition in the antisaccade task. In the gap/overlap task, the mean latency depended on the target onset time, relative to the fixation offset time. Negative onset time (gap condition) resulted in a shorter mean latency, while positive onset time (overlap condition) resulted in a longer mean latency. Notably, the results obtained by GazeParser agreed closely with those obtained by Eyelink. A two-way repeated measures ANOVA was performed to confirm these observations. For the antisaccade task, the effect of condition (antisaccade or prosaccade) was significant, $F\left( {1,5} \right) = 101.16,p < .01,\eta _{p}^{2} = .95$. The effect of measurement device was significant, $F\left( {1,5} \right)=8.99,p=.03,\eta_p^2=.64$. The interaction of these effects was not significant, $F\left( {1,5} \right)=5.40,p=.06,\eta_p^2=.52$. For the gap/overlap task, the effect of relative onset time was significant, $F\left( {4,20} \right) = 35.50,p < .01,\eta _{p}^{2} = .99$. The effects of measurement device, $F\left( {1,5} \right)=3.84,p=.11,\eta_p^2=.43$, and the interaction, $F\left( {4,20} \right)=1.26,p=.32,\eta_p^2=.20$, were not significant. Although a significant effect of measurement device was observed in the antisaccade task, we can confirm that typical results of antisaccade and gap/overlap tasks could be obtained with either GazeParser or Eyelink.

To compare the measurement of saccade amplitude as a function of device (i.e., GazeParser vs. Eyelink), the mean and *SD* of saccade amplitude in the prosaccade condition of the antisaccade task were calculated for each device. For the data recorded by Eyelink, the mean and *SD* were 4.39 and 0.60, respectively, whereas for the data recorded by GazeParser, corresponding mean and *SD* values were 4.34 and 0.43. Both mean values agreed closely with the target distance from the fixation point (4.2°). A paired *t*-test showed that mean amplitude was not significantly different between measurement devices, *t*(5) = 0.16, *p* = .88, d_*D*_ = .07).

These results show that GazeParser can measure saccade latency and amplitude reliably. However, GazeParser occasionally failed to detect gaze position in 2 participants, even though Eyelink could stably detect their gaze position. This difference probably derived from the difference in the method of eye position detection. Eyelink uses pupil image and images of four IR markers mounted on the stimulus presentation display; on the other hand, GazeParser uses both pupil image and the Purkinje image. If the detection of a participant’s Purkinje image is unstable, due to certain factors such as cornea shape and long eyelashes, the GazeParser fails to detect eye position. However, Eyelink can detect eye position even in cases with factors such as these. Improving eye position detection performance is one of the main issues that need to be addressed in future development of GazeParser.

## General discussion

Although eye movement is a useful behavioral index for studying perceptual and cognitive processes, engaging in eye movement research can be difficult because of the initial cost of recording devices and the programming time required to synchronize eye movement recording and visual stimuli. The GazeParser is an open-source library that is being developed to mitigate these difficulties. An advantage of GazeParser, when compared with other open-source eye-tracking libraries, is the ability of the software to coordinate with VisionEgg and PsychoPy. The present study showed that GazeParser was capable of recording gaze position at a rate of more than 100 samples per second. The spatial accuracy of GazeParser was reasonably high, as compared with that of other video-based eyetrackers.

The choice of a camera unit is important when constructing an eye-tracking system with GazeParser. In the present study, an OptiTrack V120 slim camera was primarily used to capture ocular images. Although this camera is available at a relatively low cost and has a good performance record, it can be used only with Microsoft Windows. Therefore, in order to use Macintosh or Linux machine as the recorder PC, other cameras are required. Currently, the cross-platform edition of GazeParser depends on OpenCV to capture a camera image. Because OpenCV supports various camera units, including low-cost USB2.0 Web cameras, these cameras might potentially be used with GazeParser. However, the performance characteristics of these cameras differ greatly, and most are not suitable for use in research. For example, some cameras cannot capture images at a constant time interval, while with other cameras, the detection of the Purkinje is unstable because of a built-in IR cut filter. Moreover, auto-adjustment of camera settings, such as gain and exposure, may have unpredictable effects on data quality. I recommend the use of quality industrial cameras with user-customizable functions. The resolution of the camera image need not necessarily be high; for example, the cameras used in this study had a resolution of only 320 × 240 or 320 × 224. Although using high-resolution images might improve spatial accuracy and precision, it might be impossible to transfer these images from the camera to the PC at a desirable sampling frequency, because limitations of transfer depend on the camera, PC, and interface unit. For example, transferring a 640 × 480 8-bit monochrome image at 120 Hz is near the practical limit of USB 2.0 interface; hardware set 1a in Experiment 1 could not transfer this image format, whereas hardware set 1b could.

One important characteristic of eye-tracking devices that was not examined in this study is the tracking range. Evaluating this range is difficult because it differs considerably across participants. If participants have long eyelashes or narrow eyes, GazeParser may encounter difficulty in pupil detection, as with other video-based eyetrackers, and, therefore, narrow the tracking range. When the eye rotates away from the center, the Purkinje image leaves from the cornea surface. In this situation, the GazeParser cannot estimate gaze position reliably. The range of eye rotation within which the Purkinje image stays on the cornea surface appears to depend on cornea shape. Individual differences in these factors affect the tracking range of GazeParser. If detection of the pupil and the Purkinje image are not disturbed by these factors, the tracking range of GazeParser reaches approximately 30° in the horizontal and 20° in the vertical directions.

GazeParser is currently under development. The main features now in development involve recording in binocular fashion, importing data recorded by other eyetrackers, and analyzing recorded data through multiple methods. Binocular recording has been tentatively implemented and is now undergoing testing. As for data importation, GazeParser can import gaze position and some event data from an Eyelink EDF file (via edf2asc.exe, which is bundled with Eyelink) and Tobii’s tab-separated text file (Tobii Technology, Ltd.). This feature is intended to provide a common interface for analyzing data recorded by these eyetrackers and GazeParser. Further improvements are necessary for enabling importation of whole data sets from these files. To support data analysis, GazeParser currently provides Python modules to detect microsaccades according to the method proposed by Engbert and Kliegl (2003), to evaluate similarity between two gaze trajectories using ScanMatch algorithm (Cristino, Mathot, Theeuwes, & Gilchrist, 2010), and to evaluate saccade trajectory curvature. Additionally, modules for other analyses will be added in the future.

In summary, GazeParser is a low-cost solution for gaze position recording. It is an open-source software that runs on Microsoft Windows, Linux, and Mac OS X, with temporal and spatial performance that is sufficient for research use.
